# A Microanalysis of Mood and Self-Reported Functionality in Stroke Patients Using Ecological Momentary Assessment

**DOI:** 10.3389/fneur.2022.854777

**Published:** 2022-05-19

**Authors:** Saskia D. Forster, Siegfried Gauggel, Rebecca Loevenich, Volker Völzke, Axel Petershofer, Petra Zimmermann, Caroline Privou, Jürgen Bonnert, Verena Mainz

**Affiliations:** ^1^Institute for Medical Psychology and Medical Sociology, University Hospital of the RWTH Aachen, Aachen, Germany; ^2^VAMED Klinik Hattingen GmbH, Hattingen, Germany; ^3^MediClin Klinik Reichshof, Reichshof-Eckenhagen, Germany

**Keywords:** ecological momentary assessment (EMA), stroke, mood, functional status, self-report

## Abstract

Post-stroke depression has been repeatedly associated with the degree of functional and cognitive impairment. The present study aimed to conduct a microanalysis on this association and examined the association between mood and self-reported functionality in 20 stroke patients (6 females, age: *M* = 59.9, *SD* = 5.2) using ecological momentary assessments (EMA), a structured diary method capturing moment-to-moment variations. Mood and self-reported functionality were recorded via a smartphone-app eight times a day for seven consecutive days during inpatient rehabilitation care. The patients answered on average to 73.2% of the received prompts. Variability in patients' responses was caused by differences both between and within patients. Multilevel regression analyses revealed that mood and self-reported functionality were significantly associated at the same point in time, but only patients' mood predicted their self-reported functionality at the next assessment point in time-lagged analyses. These results remained stable after controlling for between-person differences as patients' age, staff-ratings of their awareness of illness, and their degree of functional independence. Patients' mood appeared to affect their future ratings of their functionality but not the other way around.

## Introduction

Stroke is a leading cause of disability, creating a huge burden on affected individuals, their family members, and the healthcare system (1). In both the short-term and the long-term, many patients struggle with the physical, cognitive, and psychosocial sequelae associated with stroke. Due to the serious long-term consequences, it is not surprising that many studies have investigated these consequences and their course and treatments for them (2).

Post-stroke depression is a common complication with various negative effects on outcome. Paolucci et al. (3) reported that patients with post-stroke depression had higher levels of disability than comparable stroke patients without depression both at admission and at discharge and that they benefited less from treatment. A meta-analysis of 61 studies, which included 25,488 patients, reported that 31% of the patients (95% confidence interval: 28% to 35%) developed depression at some point up to 5 years following the stroke (4). However, prevalence of depression at 5 years after a stroke (23%; 95% confidence interval: 14% to 31%) had decreased significantly. The high prevalence, the chronicity, and the negative impact of depression on outcome explain why there is great interest in understanding the etiology of post-stroke depression (5–8).

Previous research has demonstrated that biological, psychological, and social factors play an important role in the etiology of post-stroke depression (6, 8). Premorbid mental disorders, pathophysiological stroke factors, the degree of cognitive and motor impairments together with restrictions on activity and participation have all been identified as risk factors for the development of a depressive disorder following a stroke (7, 9). One of the most consistent findings has been that post-stroke depression is positively associated with the degree of functional and cognitive impairments (6). However, the mechanisms linking stroke, impairments, and depression are still a matter of debate.

It can be assumed that the perception of a permanent disability, combined with a loss of independence, transformation of social roles, and other aversive consequences (pain, incontinence, sleep disorders, etc.) can negatively influence patients' mood. On the other hand, it can also be argued that functional impairments result from specific depressive symptoms (diminished interests, fatigue, insomnia, concentration problems), which are caused by pathophysiological factors (e.g., inflammation). Furthermore, depressed individuals may also focus on negative self-assessments more than positive ones, and this can further influence functional status ratings. In addition, one has to consider bidirectional relationships with interacting detrimental effects of impairments and mood over time.

To gain a better understanding of the potential causes, there is a need to apply more refined and more fine-grained study designs and analytic methods (10). To achieve this, ecological momentary assessment (EMA) seems to be a promising research methodology. To date, the aforementioned associations have been investigated mostly in long-term studies lasting several months or years. So far, to our knowledge the extent to which mood and functional impairment are interrelated and affect each other in the everyday perceptions of stroke patients have not been investigated in such a detailed way.

Using EMA, Forster et al. (11) demonstrated that self-reported functionality of patients with an acquired brain injury varied across the 1-week period of testing. They found that on average only 56.3% of the variability in patients' responses was due to interpatient differences, which meant that 43.7% of the variability was due to intrapatient (time-varying) differences. Not surprisingly, both patients' self-reported functionality and their self-reported mood—which was measured as a combination of energetic arousal, calmness, and valence—varied across the study period, with on average 49.2% of the variability in the self-reported mood due to interpatient and 50.8% of the variability due to intrapatient differences.

The aim of the present study was to conduct a microanalysis on the dynamic of the relationship between patients' mood and their self-reported functionality during inpatient rehabilitation. It was hypothesized that during a given point in time, patients' mood and their self-reported functionality would be related to each other. Further, it seemed plausible that patients' self-rating of their functionality would influence their mood at a later point in time through, for example, their perception of their deficits. Alternatively, it also seemed plausible that patients' mood would influence their perception of their functionality at a later point in time through, for example, their negative self-schema. These hypotheses were tested using multilevel modeling and time-lagged analyses while controlling for person-specific differences, such as patients' age, their awareness of their illness, and the severity of their impairment. It was assumed that the variance in patients' responses would be caused by both intrapatient (level 1) and interpatient (level 2) differences.

## Methods

### Participants

Participants were recruited between March 2018 and February 2020 from German-speaking inpatients who had suffered either an ischemic or hemorrhagic infarct and were being treated in one of two different neurorehabilitation hospitals; 26 of them agreed to participate. Patients were included if they were between the ages of 18 and 70 years and if their cognitive, visual, and motor functioning indicated that they were able to use a mobile device. Patients suffering from severe cognitive or motor dysfunctions and those who were aphasic or extremely disoriented were excluded.

Although 26 patients gave written informed consent, only 20 of them could be included in the data analyses. The patients who were not included did not fulfill the study's inclusion criteria (*n* = 1) despite prior screening, or they were unwilling to participate despite having given initial consent (*n* = 5). The 20 patients (6 females, 30%) who were included were aged between the ages of 47.2 and 67.7 years (mean age = 59.9 years, *SD* = 5.2). Seven patients suffered from hemiparesis, and 11 patients showed varying degrees of awareness deficits. Three patients had a prior history of stroke. Based on the clinical impressions of the treating neuropsychologists and the standardized test results, the sample was judged to be moderately impaired. The present study sample included 11 patients who had already been included in Forster et al.'s (11) study of the feasibility of using EMA with patients with various neurological disorders.

### Procedure

The neuropsychologist who treated the patients recruited them to participate in the study if they fulfilled the study's inclusion criteria. On the first day of the study, the Principal Investigator introduced the participants to the procedure. Each participant received verbal and written descriptions of the purpose and procedure of the study, and all participants' written informed consent was obtained. The ethics committee of the Medical Faculty of RWTH Aachen University approved the study (Protocol EK 306/17) in accordance with the Declaration of Helsinki. On the first day of the study, patients were provided with a smartphone, detailed instructions, and a practice trial using EMA to familiarize them with the procedure. The study was completed within 7 days, and the neuropsychologist retrieved the smartphones. Participants were not financially or otherwise compensated for their participation in the study.

### Measures

#### Awareness of Illness and Functional Independence

Their treating neuropsychologist rated patients regarding their awareness of their illness as either *intact, mildly impaired*, or *clearly impaired*. For the analyses, awareness of illness was coded as either intact *(1)* or impaired (*0*; mildly or clearly impaired). Most patients who were impaired (*n* = 11) were only mildly impaired.

The head nurse assessed patients' functional independence using the Barthel Index, which indicates how well 10 basic daily functions (e.g., eating, personal hygiene) were performed, with ratings ranging from *0* (dependent on other people) to *100* (independently of other people) (12). In this study, the Barthel Index is interpreted as an indicator for severity of impairment. Patients' mean Barthel Index was 52.5 (*SD* = 23.9, range = 10 to 95). Using Shah et al.'s (13) guidelines, a Barthel Index from 0 to 20 indicates total dependence; 21 to 60 indicates severe dependence; 61 to 90 indicates moderate dependence; and 91 to 99 indicates mild dependence.

#### Ecological Momentary Assessment

For the EMA, the software movisensXS, App version 1.3.0 (14) and Android smartphones (Motorola Moto G, third generation) were used. During seven consecutive days, patients were prompted to respond eight times per day at randomized times between 8 a.m. and 8 p.m., with at least 60 mins between any two prompts. If patients were not able to respond to a prompt, they could choose to postpone it for 5, 10, or 15 mins, or choose not to answer. To insure situation-specific answers, it was not possible to respond to the EMA more than 20 mins after the original prompt. If patients did not respond to a prompt, they were prompted up to five times to do so. Participants were informed of the prompt via vibration and auditory signals. At each prompt, patients were asked about their current activity, social context, current mood, current self-evaluation and about the frequency of their self-reflections. In the present study, we focused only on participants' assessments of their mood and their self-evaluation.

Patients' current mood was assessed using the Multidimensional Mood Questionnaire [MDMQ, (15)], which was adapted for use with EMA. In this study, we focused only on the dimension valence, which comprises two bipolar items (unwell—well [unwohl—wohl] and discontent—content [unzufrieden—zufrieden]), both of which are answered with regard to the statement “At this moment I feel…” [Im Moment fühle ich mich…] on a seven-point Likert scale ranging from 0 (e.g., unwell) to 6 (e.g., well). The dimension valence was obtained from the mean of the two bipolar items for each assessment point.

The patients' self-assessment of functionality was captured on the basis of nine functions that are typically impaired after a stroke (memory [Sich Sachen merken], functional independence [Selbstständigkeit], reliability [Zuverlässigkeit], self-confidence [Selbstbewusstsein], learning [Lernfähigkeit], understanding problems [Probleme verstehen], show insight [Einsicht zeigen], empathy [Einfühlungsvermögen], activity [Aktiv sein]). At each assessment point, patients answered the question “What (school) grade would you give yourself since the last prompt for …?” [Welche (Schul-) Note würden Sie sich seit der letzten Abfrage geben für …] in relation to the nine functions using the marking system used in schools in Germany. A grade of 1 indicates very good performance, whereas a grade of 6 indicates very poor performance. The patients' self-reported functionality was determined as the mean grade assigned on all nine functions at each assessment point. An exploratory factor analysis confirmed that a one-factor model depicted patients' self-reported functionality well. Cronbach's alpha for patients' self-reported functionality across all nine items was 0.78, indicating acceptable internal consistency.

### Data Analysis

Statistical analyses were conducted using the computing environment R, version 3.6.1 (16), and the packages *psych* [version 2.1.9; (17)], *nlme* [version 3.1-140; (18)] and *rsq* [version 2.2; (19)]. Graphics were created with the package *ggplot2* [version 3.3.5; (20)].

#### Response Rates and Degree of Variability in Patients' Mood and Self-Reported Functionality

Response rates were computed as the proportion of complete or incomplete answered prompts relative to the total number of prompts received during the entire study period. All of the following analyses were based on fully answered prompts only. Variability in patients' responses that could be attributed to both patients (level 2, between-person variability) and the individual assessments within patients (level 1, within-person variability) were calculated, using simple (intercept only), two-level models of the patients' self-reported functionality and mood. This was done to illustrate that both differences between and within patients determined the mood that patients reported and their self-reported functionality. Variability both between and within patients would support the notion of a hierarchical structure in the data, which would warrant multilevel modeling in the subsequent analyses.

#### Association Between Mood and Self-Reported Functionality

To explore the momentary and time-lagged relationship between patients' mood (valence) and their self-reported functionality, multilevel regression analyses were conducted. Separate analyses were run to test whether mood predicted patients' self-reported functionality or their self-reported functionality predicted their mood. For all analyses, random intercepts and slopes models were run. In these multilevel models, fixed effects represented the overall association between a predictor and the dependent variable, whereas the random effects represented individual differences in this association. With regard to interpretation of the results, only fixed effects are discussed, because random effects were not the focus of the research question and were included only to account for the heterogeneity in the data. To control for known between-patient differences in all of the analyses that showed significant relationships at level 1, patients' age, the rating of their awareness of their illness, and the Barthel Index were added successively as level 2 predictors. Level 1 predictors were person-mean centered and level 2 predictors, except the dummy-coded rating of patient's awareness of illness, were grand-mean centered in all analyses to obtain meaningful intercepts. To control for autocorrelation, the function *corCAR1* (nlme) was added to each multilevel regression analysis to take an autocorrelation of order one into account, when time was measured in varying intervals. $R_{adjusted}^{2}$ values were calculated following Zhang (19).

To investigate the momentary relationship between patients' mood and their self-reported functionality, separate multilevel regression analyses were performed. This was done once with patients' self-reported functionality as the dependent variable and patients' mood measured at the same point in time as the predictor variable and once with patients' mood as the dependent variable and patients' self-reported functionality measured at the same point in time as the predictor variable.

Next, time-lagged analyses were performed to examine the predictive influence that patients' self-reported functionality conducted at one point in time had on their mood at the next EMA. Conversely, in an additional analysis the predictive influence of patients' mood at one point in time on their self-reported functionality at the next EMA was also examined. In order to present the preceding time point (t-1) for both the mood and self-reported functionality-ratings, new variables were computed for the time-lagged analyses. In the time-lagged multilevel regression analyses, the level 1 predictor was entered at t-1, and the dependent variable was entered at t0. Only two consecutively answered EMAs within the same person were included in the model. Two EMAs that were not immediately consecutive or between which there was an intervening night were not considered.

## Results

### Response Rates and Degree of Variability in Patients' Mood and Self-Reported Functionality

Participants received a mean of 48 prompts (*SD* = 7.8). The total number of prompts varied among the patients, ranging from 26 to 54 (one patient was discharged early, some patients had less prompts because of technical difficulties such as a low battery). On average, patients answered 73.2% (*SD* = 16.1%) of the prompts, of which only 1.4% (*SD* = 3%) were answered incompletely. With regard to the source of the variability in patients' mood, 50.9% of the variance in patients' responses (95% CI [37.5%, 64.2%]) was due to differences between patients (i.e., level 2), whereas 49.1% (95% CI [62.5%, 35.8%]) of the variance was due to differences within patients in the different EMA assessments (i.e., level 1). Regarding patients' self-reported functionality, 73.5% of the variance in patients' responses (95% CI [61.8%, 82.6%]) was due to differences between patients (level 2), and 26.5% (95% CI [38.2%, 17.4%]) of the variance was due to differences within patients (level 1). These results indicated that further analysis using multilevel modeling was needed because of the hierarchical structure in the data.

### Associations Between Mood and Self-Reported Functionality

**Figure 1:**
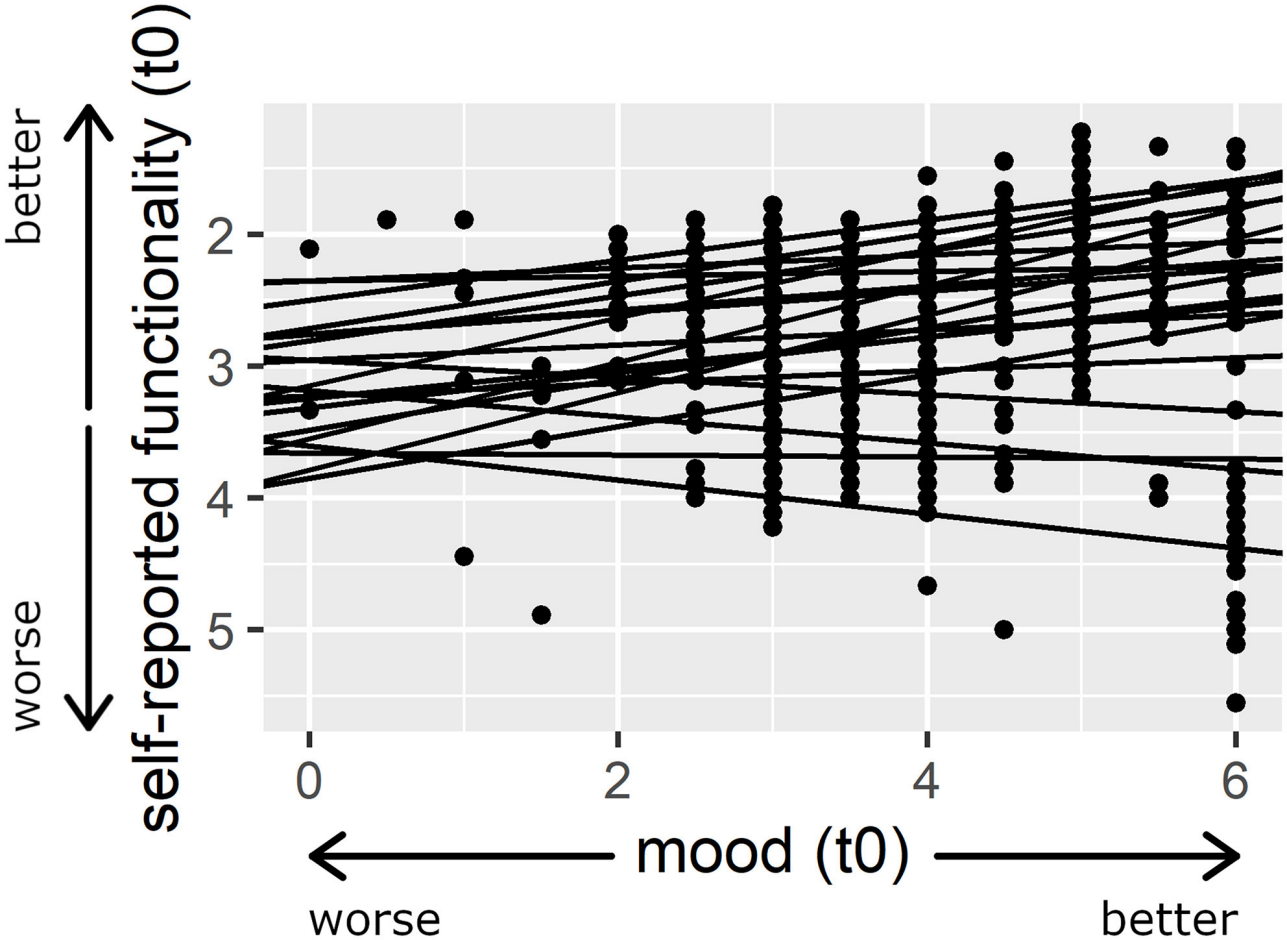
Association between patients' self-reported functionality and their current mood (t0). Larger numbers indicate better mood and smaller numbers indicate a more positive self-assessment. The regression lines illustrate the random intercepts and slopes model for patients' mood (t0) as the predictor variable and patients' self-reported functionality as the dependent variable. For presentation purposes, a model is presented here in which mood was not person-mean centered.

Multilevel regression analyses investigating the momentary associations were based on 690 observations. The multilevel regression analysis to test the relationship between patients' current mood and their current self-reported functionality yielded (a) a significant effect for patients' self-reported functionality as a level 1 predictor when mood was the dependent variable (*b* = −0.49 (β = −0.16), 95% CI [−0.8, −0.18], *SE* = 0.16, *t*_(669)_ = −3.12, *p* < 0.01), and (b) a significant effect for patients' mood as a level 1 predictor when patients' self-reported functionality was the dependent variable (*b* = −0.11 (β = −0.12), 95% CI [−0.18, −0.03], *SE* = 0.04, *t*_(669)_ = −2.8, *p* < 0.01). The significance of the level 1 predictors remained after patients' age, the rating of their awareness of illness, and their Barthel Index at level 2 had been added as additional predictor variables; see (a) Table 1 for mood as a level 1 predictor and self-reported functionality as the dependent variable, and (b) Supplementary Table 1 for self-reported functionality as the level 1 predictor and mood as the dependent variable. Figure 1 displays the level of each patient's self-reported functionality as a function of his or her current mood (t0).

**Table 1:** Random intercepts and slopes model with patients' current mood (t0) as the level 1 predictor, patients' age, awareness of illness, and the Barthel Index as the level 2 predictor and patients' self-reported functionality as the dependent variable.

***Random effects***	***SD***	**95 % CI**				
Intercept	0.62	[0.43, 0.88]				
Mood	0.14	[0.08, 0.24]				
Residual	0.36	[0.34, 0.38]				
***Fixed effects***	***b (β)***	**95 % CI**	***SE***	***df***	***t***	***p***
Intercept	2.72 (0.06)	[2.36, 3.08]	0.18	669	14.88	<0.001
Mood	−0.11 (−0.12)	[−0.18, −0.03]	0.04	669	−2.75	<0.01
Age	0.02 (0.13)	[−0.04, 0.07]	0.03	16	0.63	0.54
Awareness	−0.02 (−0.03)	[−0.58, 0.53]	0.26	16	−0.09	0.93
Barthel Index	0.01 (0.26)	[−0.004, 0.02]	0.01	16	1.36	0.19
$R_{adjusted}^{2}$ total model	0.75					
$R_{adjusted}^{2}$ fixed effects	0.13					
$R_{adjusted}^{2}$ random effects	0.62					

*Number of observations = 690*.

All time-lagged analyses were based on 468 observations because all of the non-consecutive EMAs had been excluded. On average, 91.9 mins had elapsed between two consecutive prompts (*SD* = 24.3 mins, range = 60.2 to 232.6 mins). The random intercepts and slopes model, which was used to test the predictive effects of patients' self-reported functionality as a time-lagged level 1 predictor and patients' mood as the dependent variable did not reveal a significant effect (*b* = −0.03 (β = −0.01), 95% CI [−0.35, 0.29], *SE* = 0.16, *t*_(447)_ = −0.2, *p* = 0.84). This result remained stable after patients' age, the rating of their awareness of their illness, and staff-ratings of patients' functionality (Barthel Index) had been added as level 2 predictors. The random intercepts and slopes model, which was used to test the predictive effects of patients' mood as a time-lagged, level 1 predictor and patients' self-reported functionality as the dependent variable, did, however, reveal a significant effect (*b* = −0.05 (β = −0.05), 95% CI [−0.1, −0.004], *SE* = 0.02, *t*_(447)_ = −2.15, *p* < 0.05). Also, this result remained stable after patients' age, the rating of awareness of their illness, and the Barthel Index had been added as level 2 predictors.

**Figure 2:**
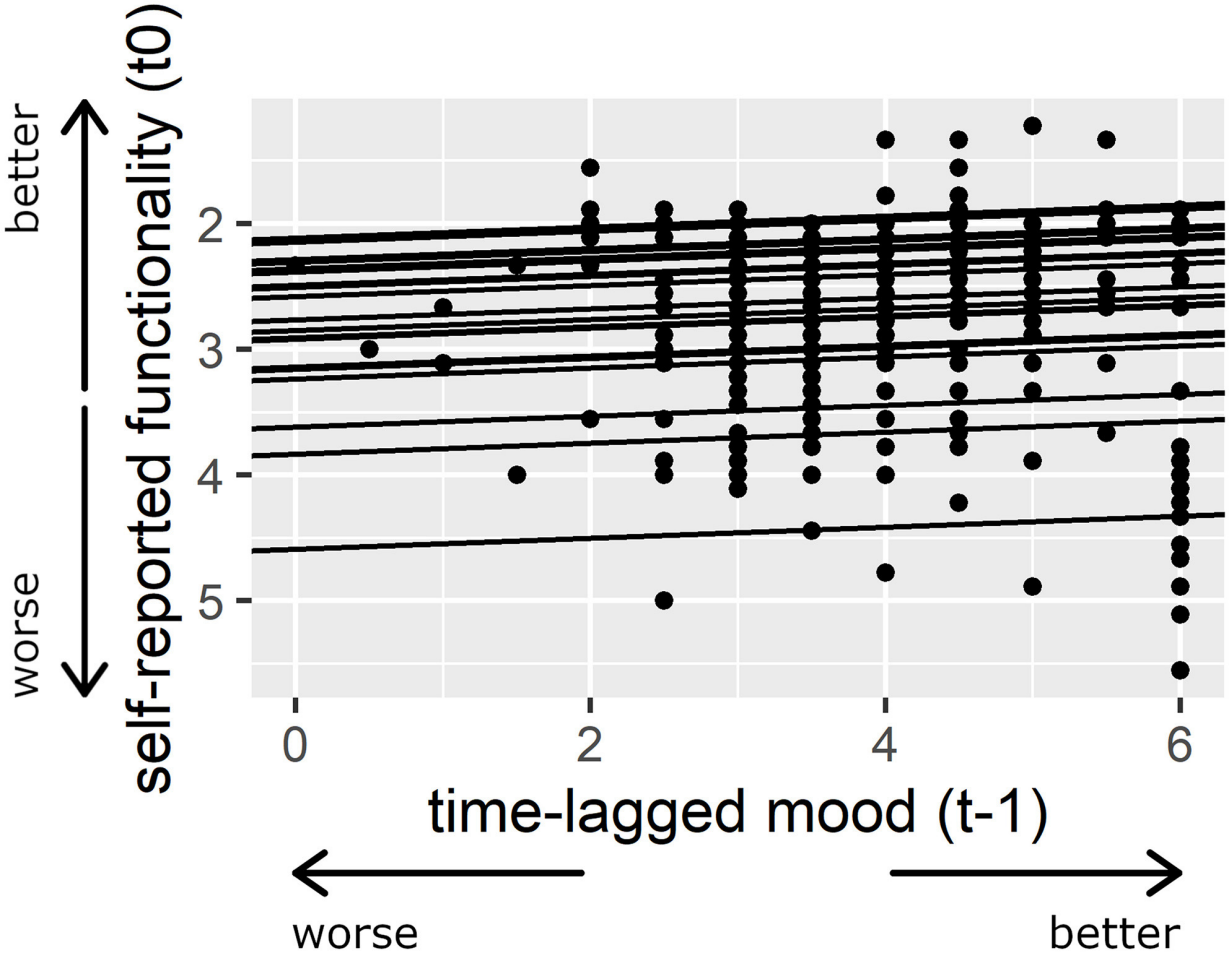
Association between patients' time-lagged mood (t-1) and their self-reported functionality. Larger numbers indicate a better mood and smaller numbers indicate a more positive self-assessment. The regression lines illustrate the random intercepts and slopes model for patients' time-lagged mood (t-1) as the predictor variable and patients' self-reported functionality as the dependent variable. For presentation purposes, a model is presented here in which mood was not person-mean centered.

The results indicate that patients' previous mood (t-1) significantly affected their subsequent rating of their functionality (t0), whereas their previous rating of their functionality (t-1) did not influence their next mood-rating (t0). The results of the random intercepts and slopes model, which included (a) the patients' mood as a level 1 predictor, (b) all of the previously named level 2 predictors, and (c) the patients' self-reported functionality as the dependent variable, are shown in Table 2 (see Supplementary Table 2 for patients' self-reported functionality as a level 1 predictor and mood as the dependent variable). Figure 2 shows the level of each patient's self-reported functionality as a function of his or her previous mood (t-1).

**Table 2:** Random intercepts and slopes model with patients' time-lagged mood (t-1) as the level 1 predictor, patients' age, awareness of illness, and the Barthel Index as the level 2 predictor and patients' self-reported functionality as dependent variable.

**Random effects**	***SD***					
Intercept	0.61					
Mood	0					
Residual	0.37					
***Fixed effects***	***b (β)***	**95% CI**	***SE***	***df***	***t***	***p***
Intercept	2.71 (0.03)	[2.34, 3.08]	0.19	447	14.41	<0.001
Mood lagged	−0.05 (−0.05)	[−0.09, −0.001]	0.02	447	−2	<0.05
Age	0.04 (0.27)	[−0.02, 0.1]	0.03	16	1.24	0.23
Awareness	−0.03 (−0.05)	[−0.63, 0.56]	0.28	16	−0.12	0.91
Barthel Index	0.01 (0.36)	[−0.002, 0.02]	0.01	16	1.75	0.10
$R_{adjusted}^{2}$ total model	0.73					
$R_{adjusted}^{2}$ fixed effects	0.10					
$R_{adjusted}^{2}$ random effects	0.63					

*Number of observations = 468, confidence intervals for the random effects could not be calculated due to the zero variance in the slopes*.

## Discussion

The aim of this study was to conduct a fine-grained analysis of the relationship between patients' mood and their self-reported functionality. This was done in order to identify the direction of the association between the two variables by examining both (a) the predictive influence of patients' self-reported functionality on their mood, and (b) the predictive influence of patients' mood on their self-reported functionality. First of all, it should be noted that patients' response rates to the EMA and the degree of variability in patients' responses was consistent with Forster et al.'s (11) results. The patients answered on average to 73.2% of the received prompts and the variance in patients' responses was caused by differences both between and within patients. Our multilevel regression analyses revealed that patients' mood and their self-reported functionality were significantly correlated with each other at a given point in time. This showed that patients who indicated that they were not in a good mood also evaluated their own functioning as poor, whereas patients who reported that they were in a good mood evaluated their own functioning better. Interestingly, the time-lagged analyses showed that patients' mood had a small but significant predictive effect on their future functionality-rating, but their self-assessment of their functionality did not affect their future mood-rating. It should also be noted that the patients' self-reported functionality also had no predictive effect on their future mood-rating when the lagged time interval was reduced to a maximum of 120 mins (number of observations = 409, *M* = 85.1 mins, *SD* = 15.8 mins). Conversely, however, the patients' mood did affect their future functionality-rating. This outcome, therefore, is relevant to the discussion about the direction of the relationship between patients' emotional reaction and their own perception of their functional impairment following stroke. The current results support the hypothesis that patients' mood affects their self-reported functionality over an extended period of time, but that their self-reported functionality does not affect their self-reported mood after an average of one and a half hours. It should be noted, however, that although post-stroke patients' mood had a significant effect on their later self-reported functionality, the magnitude of the effect was small.

Earlier research (21) emphasized the effects of mood on cognition, especially information processing. It may be that patients used their mood as metacognitive information when they evaluated their own functioning. This hypothesis is consistent with the feelings-as-information theory (22), which holds that people misattribute their pre-existing mood as reference point in their reaction to an unrelated target. Further, it has been argued that when people are in a positive mood they process information more heuristically, relying more on global information, but when they are in a negative mood they process information with a greater focus on details (23). Studies, however, have also reported that in healthy participants negative affect is associated with greater self-focused attention (24), and while they were in a negative mood, healthy participants with heightened self-focused attention reported more physical symptoms (25). Research with healthy participants has also revealed an attentional bias for mood-congruent stimuli (26). It can be conjectured either that patients misattribute their mood as indicative of their self-assessment, or when they are in a more negative mood, they experience greater self-focus and more attentional bias for mood-congruent information, and this might enhance their perception that they are impaired.

The results of the present study, however, demonstrate only that when patients are in a negative mood, they seem to be more critical about themselves and their performance, but causal explanations for this relationship remain unclear. One possibility is that patients' perception of their own functionality was also influenced by their mood-related symptoms, such as fatigue, insomnia, reduced interest, or difficulties with concentration, which were not measured in this study. In line with the biopsychosocial approach to post-stroke depression, which factors influence patients' mood in everyday life remain to be clarified. It seems reasonable to assume, however, that situational factors, such as the current social context or current activities in which the patients are engaging, influence both their mood and their assessment of their functioning.

As accounted for by the inclusion of random effects, the patients differed in most of the analyses in the effect that their mood had on their self-reported functionality (see Figure 1). The level 2 predictors that were added in the analyses might to some extent explain the differences between participants regarding the dependent variable. However, because of the small sample size, the effects of the level 2 predictors should be interpreted with caution. In addition, future studies should take cross-level interactions into account, i.e., the impact that known level 2 predictors have on the effect that mood has on individual patients' perception of their functionality. Again, a larger sample would be needed to draw firm conclusions regarding this possibility. After patients have suffered a stroke, it seems plausible that differences in their degree of awareness of their illness, neurocognitive functioning, and psychological difficulties such as depression might help to determine the effect that their mood has on their perception of their functionality.

### Limitations

As mentioned, one limitation of the current study was the small sample size, which resulted in lower power, especially for the between-participant predictors (level 2). The neuropsychologists' preselection of participants who had suffered a stroke, and who were being treated in an inpatient setting, limits the generalizability of the study's findings. In addition, because of the small sample size, subgroup analyses were not possible, for example, with regard to the patients' history of prior stroke. Further, patients' awareness of their illness was assessed through the clinical impressions of the treating neuropsychologists, because no satisfactory standardized and comprehensive assessment could be implemented in this study. Finally, we have to point out, that the self-assessment of functionality and the staff-assessment capture different aspects of functionality. In a supplemental analysis, the degree of agreement between the staff's assessment of the patients' functional independence using the Barthel Index and the self-assessment was examined. The mean estimation of the self-reported functionality using EMA was not correlated with the staff-rating (*r* = 0.33, *p* = 0.15).

### Conclusions

The finding that (a) patients' mood predicts their future perception of how well they are functioning and (b) the measured variability in these variables is of clinical relevance. Moreover, these relationships bring into question whether greater functional impairment is a risk factor for post-stroke depression, or whether the converse is true. At least within a week during patients' rehabilitation, patients' fluctuating mood seems to have a prolonged effect on their perception of their impairment, leading to a more pessimistic assessment of their own functioning when they are or have recently been in a negative mood state. Clinicians assessing patients' self-perception of their functioning during rehabilitation should take these associations into account. Further studies with larger samples are needed to achieve a better understanding of the associations that were identified in this study and other time-varying factors (e.g., social context) that might affect patients' perceptions of their functionality. Moreover, interpersonal differences in how time-varying factors affect patients' perception of their functionality deserve more research attention.

## Data Availability Statement

The anonymized raw data supporting the conclusions of this article will be made available by the authors, without undue reservation.

## Ethics Statement

The studies involving human participants were reviewed and approved by Ethics Committee of the Medical Faculty of the RWTH Aachen University, Aachen, Germany. The patients/participants provided their written informed consent to participate in this study.

## Author Contributions

The study was designed and conceptualized by VM, SF, and SG. Clinical support and recruitment of patients was done by VV, AP, PZ, CP, and JB. Data collection was conducted by RL and SF. Data analysis, statistical analyses, and visualizations were performed by SF. The manuscript was drafted by SF and SG. All authors critically reviewed and approved the manuscript.

## Funding

The study was financed by the funds of the Institute for Medical Psychology and Medical Sociology, University Hospital of the RWTH Aachen, Aachen, Germany.

## Conflict of Interest

VV and AP were employed by VAMED Klinik Hattingen GmbH, Rehabilitation Centre for Neurology, Neurosurgery, Neuropaediatrics, Hattingen, Germany. PZ, CP, and JB were employed by MediClin Klinik Reichshof, Rehabilitation Centre for Neurology and Pneumology, Reichshof-Eckenhagen, Germany. The remaining authors declare that the research was conducted in the absence of any commercial or financial relationships that could be construed as a potential conflict of interest.

## Publisher's Note

All claims expressed in this article are solely those of the authors and do not necessarily represent those of their affiliated organizations, or those of the publisher, the editors and the reviewers. Any product that may be evaluated in this article, or claim that may be made by its manufacturer, is not guaranteed or endorsed by the publisher.
