# Supplementary material for: A Microanalysis of Mood and Self-Reported Functionality in Stroke Patients Using Ecological Momentary Assessment
Source: Front Neurol. 2022 May 19;13:854777. doi: 10.3389/fneur.2022.854777 (PMC9160229; doi:10.3389/fneur.2022.854777)
Supplement: Supplementary file 1 [file Table_1.pdf]

Supplementary Table 1. Random intercepts and slopes model with patients' self-reported functionality (t0) as the level 1 predictor; patients' age, awareness of their illness, and their Barthel Index as the level 2 predictor; and patients' mood as the dependent variable.

random effects

	SD	95 % CI
intercept	0.66	[0.46, 0.94]
functionality	0.55	[0.34, 0.92]
residual	0.75	[0.70, 0.79]

fixed effects

	b (*β*)	95 % CI	*SE*	*df*	*t*	*p*
intercept	3.59 (-0.32)	[3.21, 3.97]	0.19	669	18.71	<.001
functionality	-0.45 (-0.15)	[-0.75, -0.14]	0.16	669	-2.86	<.01
age	0.02 (0.10)	[-0.03, 0.07]	0.03	16	0.80	.44
awareness	0.85 (0.75)	[0.29, 1.40]	0.26	16	3.22	<.01
Barthel Index	0.01 (0.31)	[0.003, 0.03]	0.01	16	2.62	<.05
$R^2_{adjusted}$ total model		.58				
$R^2_{adjusted}$ fixed effects		.24				
$R^2_{adjusted}$ random effects		.34				

Note. Number of observations = 690.
