# Supplementary material for: A Microanalysis of Mood and Self-Reported Functionality in Stroke Patients Using Ecological Momentary Assessment
Source: Front Neurol. 2022 May 19;13:854777. doi: 10.3389/fneur.2022.854777 (PMC9160229; doi:10.3389/fneur.2022.854777)
Supplement: Supplementary file 2 [file Table_2.pdf]

Supplementary Table 2. Random intercepts and slopes model with patients' lagged self-reported functionality (t-1) as the level 1 predictor; patients' age, awareness of illness, and their Barthel Index as the level 2 predictor; and patients' mood as the dependent variable.

random effects

	SD	95 % CI
intercept	0.67	[0.46, 0.98]
functionality	0.44	[0.20, 0.98]
residual	0.77	[0. 71, 0.83]

fixed effects

	b (β)	95 % CI	*SE*	*df*	*t*	*p*
intercept	3.63 (-0.29)	[3.22, 4.05]	0.21	447	17.19	<.001
functionality	-0.02 (-0.01)	[-0.33, 0.29]	0.16	447	-0.13	.90
age	0.03 (0.15)	[-0.04, 0.10]	0.03	16	0.98	.34
awareness	0.79 (0.70)	[0.12, 1.46]	0.32	16	2.50	<.05
Barthel Index	0.01 (0.27)	[-0.001, 0.03]	0.01	16	1.92	.07
$R^2_{adjusted}$ total model		.56				
$R^2_{adjusted}$ fixed effects		.25				
$R^2_{adjusted}$ random effects		.31				

Note. Number of observations = 468.
